# Pain chronobiology in clinical trial participants with fibromyalgia: a comparison with neuropathic pain

**DOI:** 10.1097/PR9.0000000000001307

**Published:** 2025-06-20

**Authors:** Ryan Navarro, Wilma Hopman, Ian Gilron

**Affiliations:** a Department of Anesthesiology & Perioperative Medicine, Kingston Health Sciences Centre, Queen's University, Kingston, ON, Canada; b Department of Public Health Sciences, Queen's University, Kingston, ON, Canada; c Department of Biomedical & Molecular Sciences, Queen's University, Kingston, ON, Canada; d Centre for Neuroscience Studies, Queen's University, Kingston, ON, Canada; e Providence Care Hospital, Kingston, ON, Canada

**Keywords:** Fibromyalgia, Neuropathic pain, Chronobiology, Diurnal variation, Circadian rhythms, Pain measurement

## Abstract

**Introduction:**

Diurnal pain rhythmicity is well recognized for neuropathic pain (NP) and osteoarthritis, however, less is known for fibromyalgia.

**Objective:**

We conducted secondary analyses of clinical trial data to describe pain rhythmicity in fibromyalgia compared to NP.

**Methods:**

We have compared morning–evening pain differences in fibromyalgia with that of NP by conducting exploratory analyses of data from 2 fibromyalgia trials (68 pooled participants) and 1 NP trial (55 participants). In these trials, pain intensity (0–10 scale) was rated at 8:00 am and 8:00 pm during a 7-day pretrial baseline period and throughout each trial. Analyses evaluated morning vs evening pain intensity differences for each condition as well as possible patient-specific predictors of diurnal variability.

**Results:**

Data demonstrated statistically significant morning–evening differences in both conditions such that evening pain was higher than morning pain by approximately 20% in NP (0.97 NRS, CI: 0.77–1.16) and approximately 7% in fibromyalgia (0.38 numerical rating scale (NRS), CI:0.22–0.53). The morning–evening pain intensity difference was significantly greater for NP vs fibromyalgia. In exploratory analyses of participants with fibromyalgia, older age, shorter pain duration, and more severe “hot-burning” pain rating were significantly correlated with greater morning–evening differences. In NP participants, higher body weight and higher pain interference were significantly correlated with lower morning–evening differences.

**Conclusions:**

These analyses suggest that fibromyalgia pain is slightly more intense in the evening vs morning. Although it seems less pronounced than with NP, patient subgroups with this pattern should be studied further when investigating and implementing fibromyalgia treatment interventions.

## Abstract 2

Exploratory clinical trial analyses suggest that fibromyalgia pain is more intense in the evening. This pattern should be studied further particularly when investigating fibromyalgia interventions.

## 1. Introduction

Fibromyalgia (FM) is a complex chronic pain disorder characterized by widespread pain, fatigue, sleep disturbances, and cognitive dysfunction, affecting approximately 2% to 8% of the population.^7,31,37^ Despite its prevalence, the underlying pathophysiology of FM remains poorly understood, although it is increasingly recognized as involving central sensitization, with amplified pain signalling in the central nervous system.^7^ This heightened pain perception has been linked to imbalances in neurotransmitters, such as elevated glutamate and reduced gamma-aminobutyric acid (GABA) and dysregulation of the hypothalamic–pituitary–adrenal axis.^33^ In addition, small fiber pathology has been reported in patients suffering from FM as manifested by decreased intraepidermal nerve fiber density.^27,34,36^ Fibromyalgia pain shares clinical features with neuropathic pain (NP), including hyperalgesia and allodynia, but likely differs in its underlying mechanisms and is classified as nociplastic pain—arising from altered nociception without clear evidence of tissue damage.^20,21^

Temporal patterns of pain have been described in a number of painful conditions, often showing, on average, predictable variation throughout the circadian cycle.^4,16,18,19,32^ For instance, inflammatory pain conditions such as arthritis, osteoarthritis, and postsurgical pain show peak pain intensity levels in the early morning,^2,3,22^ whereas peripheral neuropathic pain conditions exhibit higher pain levels in the evening.^12,18,28^

By contrast, the circadian variation in nociplastic pain, such as FM, remains underexplored. A better understanding of circadian pain patterns in FM could help optimize interventions and improve pain management strategies. The limited existing data on diurnal rhythms in FM (n = 21) suggest moderate variation in pain throughout the day, although more robust studies are needed to confirm these findings.^1^ Identifying clinical predictors of diurnal pain rhythmicity in FM may also provide insights into its mechanisms^32^ and inform time-targeted interventions.^35^

Thus, using data from the IMPALA (Innovations in the Management of Musculoskeletal Pain With Alpha-Lipoic Acid)^13^ and CADENCE (Combination Analgesic Development for Enhanced Clinical Efficacy)^15^ trials in participants with FM and the PAIN CARE (Pain Improvement With Novel Combination Analgesic Regimens)^14^ trial in participants with neuropathic pain, we conducted a series of secondary analyses with the goals of (1) characterizing diurnal pain patterns in FM, (2) identifying the association between various clinical predictors and diurnal pain rhythmicity in FM, and (3) comparing diurnal pain patterns in FM with those observed in neuropathic pain.

## 2. Methods

This investigation involved exploratory secondary analyses of data from 3 previously published randomized controlled crossover trials of patients with fibromyalgia^13,15^ (IMPALA and CADENCE trials) and neuropathic pain^14^ (PAIN CARE trial). These trials all received approval from the Queen's University Research Ethics Board, were registered with the International Standard Randomised Controlled Trial Number (ISRCTN) registry, and involved participants who experienced daily moderate–severe pain for at least 3 months and with no evidence of major psychiatric or substance abuse disorder.

## 3. Study 1: alpha-lipoic acid trial for fibromyalgia (innovations in the management of musculoskeletal pain with alpha-lipoic acid trial)

Study 1 was a single-centre, double-blind, randomized, 2-period crossover trial (5 weeks per treatment period) comparing alpha-lipoic acid (ALA) with placebo in 27 adult patients with fibromyalgia.^13^ At the beginning of each period, participants received 1 set of either ALA 300 mg tablets or placebo (lactose capsules) to be taken 30 minutes before meals. The primary outcome was the mean daily “average” pain intensity experienced while on the maximal tolerated dose (MTD) of ALA or placebo during week 4 (days 22–28). This was determined from participants' ratings of their “average pain over the past 24 hours” completed in patient diaries every morning using a numerical rating scale from 0 to 10. In addition, pain severity was assessed at 8:00 am and 8:00 pm each day. Baseline pretrial assessments included age, sex/gender, body weight, Brief Pain Inventory, 2016 Fibromyalgia Diagnostic Criteria indices (Widespread Pain Index, Total Fibromyalgia Score, severity of fatigue, cognitive issues, and waking unrefreshed), Beck Depression Inventory-2 scores, Medical Outcomes Study Sleep Survey, duration of chronic pain, and concomitant pain medications. Secondary on-trial outcomes included Medical Outcomes Study Sleep Scale, Patient Global Impression of Change, and Brief Pain Inventory (BPI). These outcomes were assessed at baseline and during week 4 of each treatment period.

## 4. Study 2: alpha-lipoic acid–pregabalin combination trial for fibromyalgia (combination analgesic development for enhanced clinical efficacy trial)

Study 2 was designed as follows: (1) there were 3 treatment periods and 3 trial treatments: pregabalin, ALA, or pregabalin–ALA combination; (2) patients were randomized (double-blind, according to a balanced Latin square design) to 1 of 6 possible sequences of these 3 treatments; (3) each treatment period was 6 weeks long with 24 days of dose titration, 1 week at MTD, 1-week dose taper, and 4-day washout.^15^ A total of 41 participants were enrolled, 28 participants completed ≥2 study periods, and 24 participants completed all 3 periods of the trial. Baseline pretrial assessments included age, sex/gender, body weight, Brief Pain Inventory, 2016 Fibromyalgia Diagnostic Criteria indices (Widespread Pain Index, Total Fibromyalgia Score, severity of fatigue, cognitive issues and waking unrefreshed), short-form McGill Pain Questionnaire (SF-MPQ), Beck Depression Inventory-2 scores, Medical Outcomes Study Sleep Survey, duration of chronic pain, and concomitant pain medications. In addition to the on-trial secondary outcomes measured in the IMPALA trial, the SF-MPQ was also administered.

## 5. Study 3: alpha-lipoic acid–pregabalin combination trial for neuropathic pain (PAIN-CARE trial)

Study 3 followed the same design as the CADENCE trial but included patients with neuropathic pain rather than those with fibromyalgia.^14^ For inclusion of participants with neuropathic pain, we followed the updated system for grading probable or definite neuropathic pain as reported by the Neuropathic Pain Special Interest Group of the International Association for the Study of Pain, and participants were assessed with electromyography, nerve conductions studies, and other special testing as guided by clinical history and examination. Eligible participants were required to have a preliminary score of 3 or higher on the “DN4 interview” 5 during telephone interview, with a final DN4 score of 4 or higher on clinic evaluation and physical examination. A total of 55 participants were enrolled, 46 participants completed ≥2 treatment periods and 44 participants completed all 3 treatment periods. Baseline pretrial assessments included age, sex/gender, body weight, Brief Pain Inventory, SF-MPQ, Beck Depression Inventory-2 scores, Medical Outcomes Study Sleep Survey, duration of chronic pain, and concomitant pain medications. The same on-trial primary outcome was measured in all 3 studies. Compared with the CADENCE trial, the on-trial secondary outcomes measured during PAIN-CARE differed only in replacing the Fibromyalgia Impact Questionnaire with the Neuropathic Pain Symptom Inventory.

In all 3 of these trials, participants were allowed to continue opioids (90 mg morphine equivalents), antidepressants (tricyclic, serotonin selective reuptake inhibitor, serotonin–norepinephrine reuptake inhibitor), nonsteroidal anti-inflammatory drugs, or acetaminophen at a steady dose throughout the entire study. Any cognitive–behavioral therapy or exercise programs could continue if scheduled evenly across all treatment periods. Participants were instructed not to start new cognitive–behavioral therapy or exercise programs after study initiation and to avoid any procedural therapies (eg, nerve blocks or acupuncture) during the entire study.

### 5.1. Data analyses

Data for the current exploratory analyses were compiled in Excel and imported into IBM SPSS (version 29.0 for Windows, Armonk, NY, 2023) for statistical analysis. Data were initially analyzed descriptively, and the Shapiro–Wilk test was used to assess the underlying distribution of the continuous data. The 95% confidence intervals (CIs) were calculated by IBM SPSS by first determining the sample mean and standard deviation, then using those values, along with the sample size and the appropriate critical value (1.96), to compute the margin of error. The CIs were then calculated by adding and subtracting the margin of error from the sample mean. Initially, the difference between morning and afternoon pain was calculated and averaged over the 7 days; between-group comparisons were made using the 1-way ANOVA and the Kruskal–Wallis tests for 3-group comparisons, and independent samples *t* tests and the Mann–Whitney *U* tests for 2-group comparisons. The data for the days of the week were then compared using repeated-measures ANOVA, with morning/afternoon as one factor and day of the week as a second factor. To further explore the am/pm data, paired *t* tests and the Wilcoxon signed-rank test were used to compare the individual days. To assess potential clinical predictors of diurnal variation in pain, independent samples *t* tests, Mann–Whitney *U*, and Spearman correlation tests were used to assess the association with age, sex, weight, and several pain-related measures (short-form McGill Pain Questionnaire [SF-MPQ—for CADENCE and PAIN-CARE only], duration of chronic pain, concomitant pain medications, Widespread Pain Index, Total Fibromyalgia Score, severity of fatigue, cognitive issues and waking unrefreshed [for IMPALA and CADENCE only], BPI activity, mood, work, social, sleep, and enjoyment, as well as BDI-2 and MOS-sleep scores). A *P*-value of <0.05 was used as the cut-point for statistical significance, and no adjustment was made for multiple comparisons.

## 6. Results

**Figure 1.**
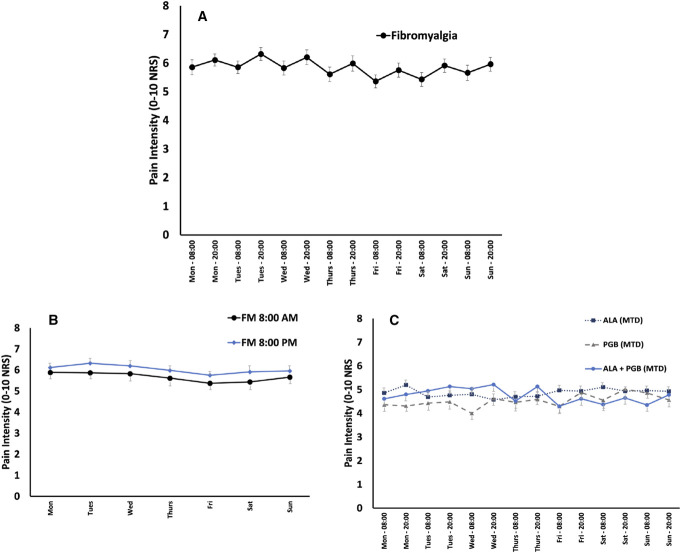
Diurnal pain rhythmicity in fibromyalgia. (A) Baseline diurnal pain rhythmicity in clinical trial participants with fibromyalgia. Error bars represent standard error of the mean. (B) Baseline morning vs evening pain intensity differences in clinical trial participants with fibromyalgia. Repeated-measures ANOVA showed a statistically significant effect of time of day (*P* < 0.001) but not for day of the week (*P* = 0.98). (C) On study diurnal pain rhythmicity in clinical trial participants with fibromyalgia during treatment with maximally tolerated doses of alpha-lipoic acid (ALA), pregabalin (PGB), and ALA + PGB combination.

**Figure 2.**
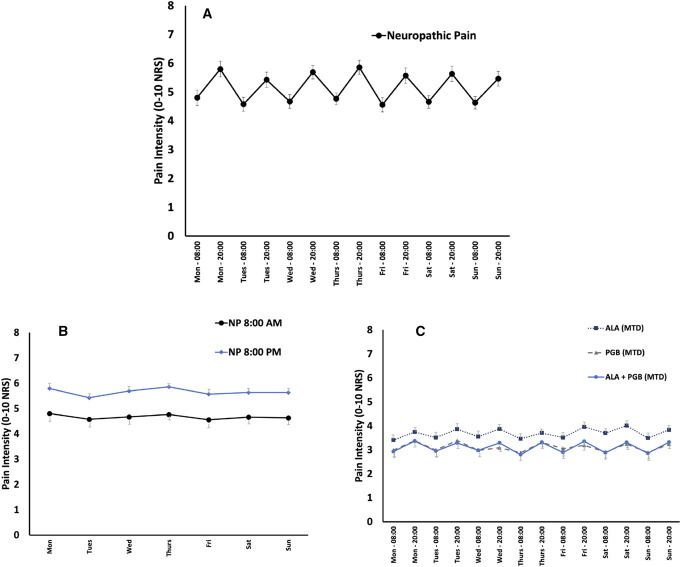
Diurnal pain rhythmicity in neuropathic pain. (A) Baseline diurnal pain rhythmicity in clinical trial participants with neuropathic pain. Error bars represent standard error of the mean. (B) Baseline morning vs evening pain intensity differences in clinical trial participants with neuropathic pain. Repeated-measures ANOVA showed a statistically significant effect of time of day (*P* < 0.001) but not for day of the week (*P* = 0.99). (C) On study diurnal pain rhythmicity in clinical trial participants with neuropathic pain during treatment with maximally tolerated doses of alpha-lipoic acid (ALA), pregabalin (PGB), and ALA + PGB combination.

Participants included in these exploratory analyses are summarized in Table 1, and additional participant details are reported in the publications of each trial.^12–14^ The diurnal and circaseptan patterns of pain intensity at pretrial baseline are shown in Figures 1 and 2, for patients with fibromyalgia (pooled from the IMPALA, n = 27, and CADENCE, n = 41, trials) and those with neuropathic pain (PAIN-CARE trial, n = 55), respectively.

**Table 1:** Participants included in exploratory analyses of diurnal pain rhythmicity.*

Clinical trial	Pain-related diagnoses	Participants enrolled	Concomitant medications (taken at constant doses throughout the trial), n (%)
IMPALA^15^	Fibromyalgia^2^	27	Acetaminophen 9 (33); NSAIDs/Cox-2I 11 (41); Codeine 1 (4); Tapentadol 1 (4); Methocarbamol 3 (11); Oxycodone 1 (4); Pregabalin 1 (4); Nortriptyline 2 (7); Amitriptyline 1 (4); Duloxetine 2 (7)
CADENCE^16^	Fibromyalgia^2^	41	Duloxetine 12 (29); Acetaminophen 8 (20); Tricyclic antidepressant 5 (12); NSAID/COX-2I 4 (10); Cannabinoid 4 (10); Magnesium 3 (7); Codeine 1 (2); Cyclobenzaprine 1 (2)
PAIN-CARE^17^	Neuropathic pain [Diabetic peripheral neuropathy^20^; Idiopathic small fiber neuropathy^19^; Chemotherapy-induced peripheral neuropathy^9^; Charcot–Marie–Tooth disease^3^; Posttraumatic neuropathy^2^; Postherpetic neuralgia^1^]	55	Acetaminophen 9 (17); Tricyclic antidepressant/serotonin–norepinephrine; reuptake inhibitor antidepressant 6 (11); Nonsteroidal anti-inflammatory drug 4 (7); Cannabinoid 3 (6); Codeine 1 (2); Tapentadol 1 (2)

* Additional participant details are reported in the publications of each cited trial.

IMPALA, innovations in the management of musculoskeletal pain with alpha-lipoic acid; NSAID, nonsteroidal anti-inflammatory drug; CADENCE, combination analgesic development for enhanced clinical efficacy.

In both pain conditions, evening pain intensity was statistically significantly higher than morning pain (8:00 am to 8:00 pm, *P* < 0.001), indicating an increasing diurnal pattern of pain intensity (Figs. 1 and 2). In the combined FM cohort, evening pain (0–10 numerical rating scale) was on average 0.38 points (CI: 0.22–0.53) or approximately 7% higher than morning pain. In the NP cohort, evening pain 0.97 points (CI: 0.77–1.16) or approximately 20% higher than morning pain. In both pain conditions, no significant circaseptan variation in pain was observed across the days of the week.

**Figure 3.**
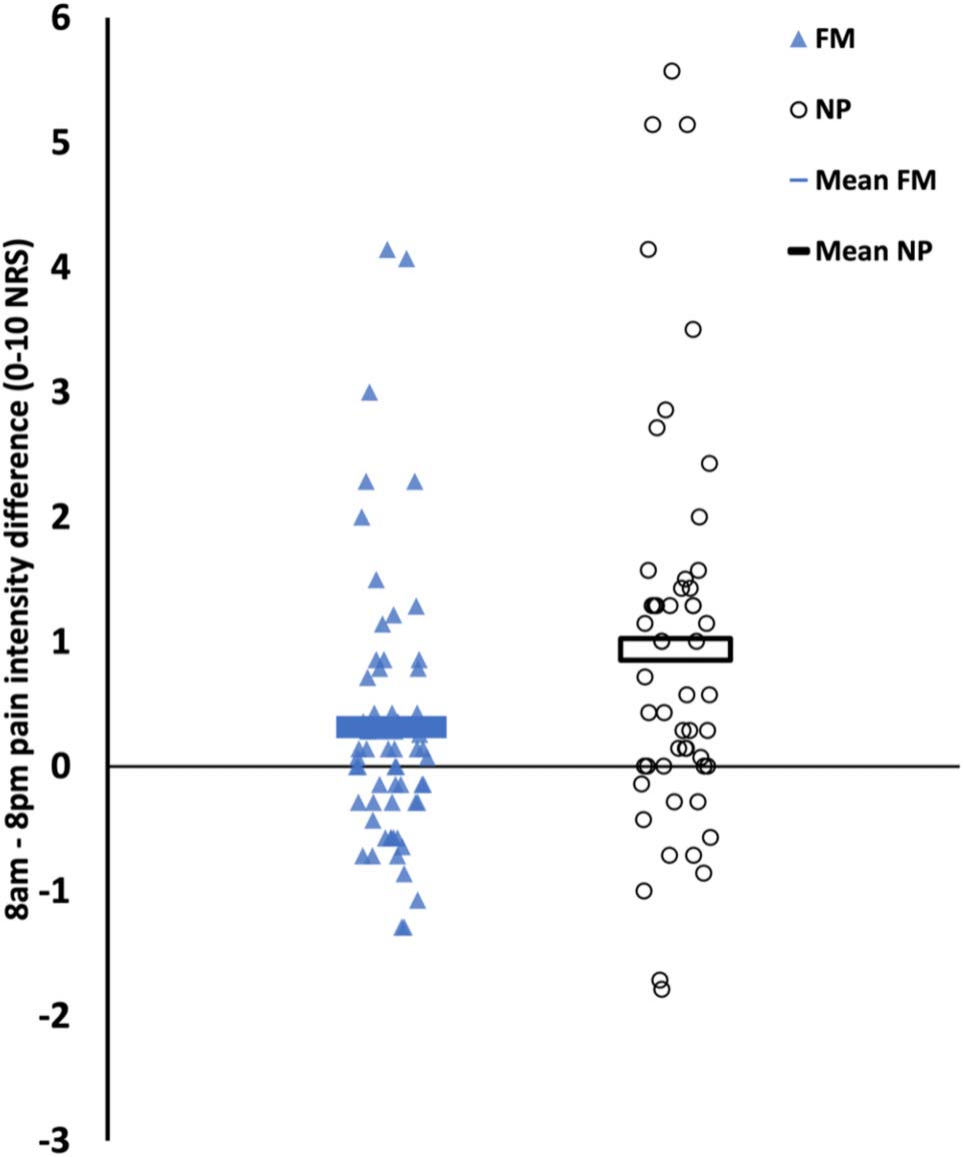
Differences in diurnal pain rhythmicity between fibromyalgia and neuropathic pain. Morning vs evening pain intensity differences were significantly greater for neuropathic pain (right) than for fibromyalgia (left) (*P* = 0.025, Mann–Whitney *U* test).

Comparatively, the neuropathic pain cohort exhibited a greater degree of diurnal variation in pain scores than the fibromyalgia group (Fig. 3). Specifically, there was a larger average morning–evening pain difference in the neuropathic pain cohort (FM vs NP, 8:00 am to 8:00 pm difference, *P* = 0.025).

### 6.1. Effects of pregabalin and/or alpha-lipoic acid on morning–evening pain differences

Figure 1C (fibromyalgia) and 2C (neuropathic pain) show the diurnal and circaseptan patterns of pain intensity in participants during treatment with maximally tolerated doses of pregabalin, alpha-lipoic acid, and their combination. In participants with FM treated with ALA alone, the morning–evening pain differences in pain intensity across the 7 days of the MTD week of the trial were no longer statistically significant (*P* = 0.9). In participants with NP treated with ALA alone, evening pain intensity across the 7 days of the MTD week of the trial was statistically significantly higher than morning pain (*P* < 0.001). In participants with FM treated with pregabalin alone, the morning–evening pain differences in pain intensity across the 7 days of the MTD week of the trial failed to reach statistical significance (*P* = 0.07). In participants with NP treated with pregabalin alone, evening pain intensity across the 7 days of the MTD week of the trial was statistically significantly higher than morning pain (*P* < 0.001). In participants with FM treated with pregabalin–ALA combination, evening pain intensity across the 7 days of the MTD week of the trial was statistically significantly higher than morning pain (*P* < 0.001). In participants with NP treated with pregabalin–ALA combination, evening pain intensity across the 7 days of the MTD week of the trial was statistically significantly higher than morning pain (*P* < 0.001).

### 6.2. Clinical predictors of diurnal rhythmicity

These exploratory analyses evaluated potential clinical predictors of diurnal pain variation, focusing on pretrial baseline age, sex/gender, body weight, Brief Pain Inventory, short-form McGill Pain Questionnaire (SF-MPQ—for CADENCE and PAIN-CARE only), 2016 Fibromyalgia Diagnostic Criteria Scores (for IMPALA and CADENCE only), Beck Depression Inventory-2 scores, Medical Outcomes Study Sleep Survey, duration of chronic pain, concomitant pain medications, and several on-trial pain-relevant secondary outcome measures (BPI interference items, BDI-2, and MOS-sleep scores). In the FM cohort, age (*P* = 0.032), duration of chronic pain (*P* = 0.017), and severity of SF-MPQ “hot-burning” pain (*P* = 0.003) were significantly correlated with diurnal pain differences such that older age, shorter duration of chronic pain, and more severe “hot-burning” pain are linked to greater morning–evening differences. In the NP cohort, body weight (*P* = 0.013), pain interference with walking (*P* = 0.044), and pain interference with work (*P* = 0.004) correlated with diurnal variation, where higher body weight and pain interference were associated with lower morning–evening differences. Despite these findings, the overall correlations were not strong enough to support the development of a reliable statistical model for predicting diurnal variation based on these predictors.

## 7. Discussion

### 7.1. Summary of findings

This investigation provides novel evidence that FM exhibits diurnal pain rhythmicity such that pain is significantly more intense in the evening compared with morning. This pattern reflects the group average of these participants with FM; however, it should be acknowledged—as seen in Figure 3—that proportions of participants with FM and also with neuropathic pain actually exhibit an inverse pattern with higher morning pain. In contrast to these most recent findings, previous studies have yielded conflicting results.^19^ A previous study by Bellamy et al. reported that FM pain was only slightly worse, approximately 4 points higher on a 0 to 100 VAS scale, in the morning vs the evening, based on a small cohort of 21 participants.^1^ A more recent study of participants with fibromyalgia examined—over different time periods in the day—the correlation between pressure pain threshold and 6‐sulfatoxymelatonin, a melatonin metabolite excreted in the urine, but did not specifically report the temporal pattern of pain intensity.^6^ Our results of higher evening pain, coming from sample size larger than that of Bellamy et al., demonstrated that FM pain follows a pattern of higher evening pain similar to neuropathic pain (NP). This suggests a distinction from diurnal pain patterns seen with inflammatory conditions such as arthritis.^2,19,22^

The comparison between FM and NP also provided valuable insights. Although both conditions demonstrated significant diurnal pain rhythmicity, the amplitude of the morning-to-evening difference was more pronounced in NP compared with FM (8:00 am to 8:00 pm difference, *P* = 0.025). Neuropathic pain, associated with diabetic peripheral neuropathy (DPN) and postherpetic neuralgia (PHN), has been previously reported to exhibit a diurnal pattern, characterized by increasing pain intensity throughout the day, peaking in the evening.^12,18,28^ The similarity in diurnal patterns between FM and NP imply that fibromyalgia and neuropathic pain may involve common mechanism that underly diurnal variation. Regarding the observed diurnal pain variations (for neuropathic pain, on average, 20% more intense in the evening and, for fibromyalgia, on average, 7% more intense in the evening), the clinical relevance of these morning–evening pain differences will obviously vary across individuals depending on the actual magnitude of morning–evening pain difference. Thus, future studies could more directly address this question with self-report assessments of participant ratings of the subjective impact of diurnal pain variation and to evaluate its association with other clinical factors.

### 7.2. Potential mechanisms of diurnal pain rhythmicity

One hypothesis to explain these findings is that increasing pain intensity throughout the day is driven by central sensitization, a hallmark of FM, which may amplify pain signals, for example, through temporal summation.^16,28^ The potential for temporal summation as a mechanism to explain why pain may become more intense throughout the day is that repeated external physical stimulation (eg, skin stimulation by clothing) or cumulative physical activity (eg, walking and working) progressively “winds up” nociceptive processing to maximal levels by the end of the day. However, for this to be a plausible mechanism for higher evening pain, these daytime nociceptive inputs would need to be more impactful than mechanical nociceptive inputs that occur during sleeping hours (eg, from bed-related movement and pressure). The clinical trials yielding the data presented here were not designed to evaluate this mechanism; however, future studies that include patient-level measures of physical activity (eg, ecological momentary assessment and/or actigraphy) would help address this question. Central sensitization involves an imbalance in excitatory and inhibitory neurotransmission, potentially driven by elevated levels of excitatory neurotransmitters such as glutamate, which could vary diurnally and lead to progressively increased pain in the evening.^7,32^ This suggests that the underlying hyperexcitability of the central nervous system may interact with circadian rhythms to influence the timing of peak pain intensity in FM. The dysregulation of the hypothalamic–pituitary–adrenal (HPA) axis also presents a plausible mechanism for the diurnal variation observed in FM. For example, evidence from neuropathic mice implicates a role for glucocorticoids acting at spinal microglia to enhance pain hypersensitivity after nerve injury.^23^ Patients with FM are known to exhibit altered HPA axis function, including abnormal cortisol secretion patterns, which could contribute to increased pain sensitivity later in the day.^7,24,33^ Reduced cortisol levels in the evening may impair the body's ability to modulate pain effectively, leading to heightened pain perception at this time.^9^ Moreover, stress-related activation of the HPA axis may exacerbate the central sensitization seen in FM, further contributing to evening pain peaks.^8^

Peripheral mechanisms may also play a role in the diurnal pain rhythmicity of FM. Small fiber pathology (decreased intraepidermal nerve fibre density), which has been identified in patients with neuropathic pain, and also, in subsets of patients with FM, could contribute to pain variation throughout the day due to accumulated peripheral input and reduced nerve fiber function over time.^25,27,36^ This suggests that both central and peripheral factors may converge to produce the diurnal pain pattern observed in FM. In painful conditions, reduced opioid receptor availability or impaired opioid signaling could contribute to circadian pain fluctuations.^10,30^ In fibromyalgia, these fluctuations could contribute to reduced pain modulation during the evening hours.^17^ This mechanism may partially explain the observed similarity in diurnal pain patterns between FM and NP. Another potential mechanism involves the role of endogenous opioids, which are crucial for modulating pain and have been shown to exhibit circadian variation. Beta-endorphin levels are highest in the morning and decline throughout the day, which may lead to reduced endogenous pain inhibition in the evening.^30^

Although NP is characterized by well-established diurnal pain rhythmicity, primarily influenced by peripheral nerve damage, FM's diurnal pattern is likely a result of a more complex interplay between central sensitization, descending inhibitory tone, neuroendocrine dysregulation, and possibly peripheral contributions.^5^ The similarity in diurnal patterns between FM and NP suggests that common pathways, such as altered neurotransmission and circadian modulation of pain, could underlie both conditions. However, the less pronounced amplitude of diurnal variation in FM compared with NP highlights that while similar mechanisms may be involved, their contributions and interactions likely differ in magnitude or timing.

### 7.3. Clinical predictors of diurnal rhythmicity

Among a variety of possible factors including age, sex, weight, and several measures of pain interference with activity, mood, work, social, sleep, and enjoyment (Brief Pain Inventory), as well as mood (BDI-2) and sleep (MOS-sleep), the only emerging predictors of diurnal pain rhythmicity in fibromyalgia trial participants were age, duration of chronic pain, and severity of SF-MPQ “hot-burning” pain descriptor ratings such that older age, shorter duration of chronic pain, and more severe “hot-burning” were associated with greater morning–evening pain differences and overall correlations were not strong enough to support the development of a reliable statistical model for predicting diurnal variation based on these predictors. The significant association between Brief Pain Inventory interference with work and diurnal pain variation in neuropathic pain trial participants is relevant to our PAIN-CARE trial cohort that included participants who worked in the home (eg, housework duties), in a workplace setting, or both. Of note, higher pain interference with work was predictive of smaller morning–evening pain differences. This could potentially be explained by an adaptive pattern whereby individuals with high pain interference limit their work activity and thus reduce temporal summation of activity-related increases of nociception throughout the day. However, regardless of these observations of possible predictors of morning–evening pain differences, it should be reiterated that these come from secondary exploratory analyses thus requiring further study and replication.

### 7.4. Limitations and future research needs

This investigation should be considered in light of some limitations. First, these observations come from exploratory analyses of clinical trials that were designed for another purpose and which may not have been statistically powered for the objective of evaluating diurnal pain rhythmicity. In addition, the trials from which these data were obtained only provided pain intensity ratings at 8 am and 8 pm and thus a limited characterization of the pain intensity profile over the course of a day. As summarized in Table 1, participants were required to take concomitant pain medications regularly throughout the trial and not on an “as needed” basis. However, it is possible, for at least some participants, that the dosing schedule of one or more concomitant medications were being taken asymmetrically throughout the day in such a way that could contribute to the observed morning–evening pain difference. Thus, future investigations to describe the diurnal rhythmicity of pain in fibromyalgia should involve appropriately powered, likely larger cohort studies that assess pain intensity over multiple timepoints throughout the day.^26^

### 7.5. Clinical and research implications

The novel finding that FM displays diurnal rhythmicity with pain higher in the evening—similar to NP—has potential clinical implications. Understanding that FM pain may be higher in the evening provides a basis for optimizing the timing of analgesic treatments to maximize their effectiveness during periods of peak pain. Such time-targeted chronotherapy has been evaluated in other painful conditions such as rheumatoid arthritis^4,35^ and could potentially also be useful for fibromyalgia, eg, by administering sedating analgesics later in the day when pain is highest and avoiding them earlier in the day so as to minimize daytime somnolence.^11^ The functional impact of evening pain peaks, particularly in patients with NP, underscores the importance of addressing diurnal pain variation to enhance patients' quality of life and their ability to engage in daily activities. Patients may benefit from strategies such as scheduled rest periods or tailored exercise plans to manage peak pain times effectively, thereby improving overall functioning and well-being. These observations also highlight important research implications in the setting of clinical pain investigations. In particular, recognizing that many participants exhibit diurnal pain rhythmicity points to the need to either: (1) measure present pain intensity at multiple specific timepoints for a comprehensive assessment of pain throughout the day and to evaluate its variability; or (2) to measure “average” daily pain whereby research participants provide their own assessment of global pain experience over the course of the preceding day.^29^

## 8. Conclusion

These exploratory analyses suggest that fibromyalgia pain is generally more intense in the evening vs morning. Although it seems less pronounced than with NP, this pattern should be studied further and also recognized when investigating and implementing fibromyalgia treatment interventions.

## Disclosures

R.N. declares no conflicts of interest. W.M.H. declares no conflicts of interest. I.G. has received support from Vertex and Combigene and has received grants from the Canadian Institutes of Health Research, Physicians' Services Incorporated Foundation, and Queen's University.
